# Heart rate reduction after genetic ablation of L-type Ca_v_1.3 channels induces cardioprotection against ischemia-reperfusion injury

**DOI:** 10.3389/fcvm.2023.1134503

**Published:** 2023-08-01

**Authors:** Viviana Delgado-Betancourt, Kroekkiat Chinda, Pietro Mesirca, Christian Barrère, Aurélie Covinhes, Laura Gallot, Anne Vincent, Isabelle Bidaud, Sarawut Kumphune, Joël Nargeot, Christophe Piot, Kevin Wickman, Matteo Elia Mangoni, Stéphanie Barrère-Lemaire

**Affiliations:** ^1^ Institut de Génomique Fonctionnelle, Université Montpellier, CNRS, INSERM, Montpellier, France; ^2^ LabEx Ion Channel Science & Therapeutics (ICST), Université de Nice, Valbonne, France; ^3^ Department of Physiology, Faculty of Medical Science, Naresuan University, Phitsanulok, Thailand; ^4^ Biomedical Engineering Institute (BMEi), Chiang Mai University, Chiang Mai, Thailand; ^5^ Département de Cardiologie Interventionnelle, Clinique du Millénaire, Montpellier, France; ^6^ Department of Pharmacology, University of Minnesota, Minneapolis, MN, United States

**Keywords:** heart rate, myocardial infarction, cardioprotection, ischemia-reperfusion injury, heart rate reduction, cav1.3 calcium channel, genetic model

## Abstract

**Background:**

Acute myocardial infarction (AMI) is the major cause of cardiovascular mortality worldwide. Most ischemic episodes are triggered by an increase in heart rate, which induces an imbalance between myocardial oxygen delivery and consumption. Developing drugs that selectively reduce heart rate by inhibiting ion channels involved in heart rate control could provide more clinical benefits. The Ca_v_1.3-mediated L-type Ca^2+^ current (*I_Cav1.3_*) play important roles in the generation of heart rate. Therefore, they can constitute relevant targets for selective control of heart rate and cardioprotection during AMI.

**Objective:**

We aimed to investigate the relationship between heart rate and infarct size using mouse strains knockout for Ca_v_1.3 (*Ca_v_1.3^−/−^*) L-type calcium channel and of the cardiac G protein gated potassium channel (*Girk4^−/−^*) in association with the funny (f)-channel inhibitor ivabradine.

**Methods:**

Wild-type (WT), *Ca_v_1.3^+/−^*, *Ca_v_1.3^−/−^* and *Girk4^−/−^* mice were used as models of respectively normal heart rate, moderate heart rate reduction, bradycardia, and mild tachycardia, respectively. Mice underwent a surgical protocol of myocardial IR (40 min ischemia and 60 min reperfusion). Heart rate was recorded by one-lead surface ECG recording, and infarct size measured by triphenyl tetrazolium chloride staining. In addition, *Ca_v_1.3^−/−^* and WT hearts perfused on a Langendorff system were subjected to the same ischemia-reperfusion protocol *ex vivo*, without or with atrial pacing, and the coronary flow was recorded.

**Results:**

*Ca_v_1.3^−/−^* mice presented reduced infarct size (−29%), while *Girk4^−/−^* displayed increased infarct size (+30%) compared to WT mice. Consistently, heart rate reduction in *Ca_v_1.3^+/−^* or by the f-channel blocker ivabradine was associated with significant decrease in infarct size (−27% and −32%, respectively) in comparison to WT mice.

**Conclusion:**

Our results show that decreasing heart rate allows to protect the myocardium against IR injury *in vivo* and reveal a close relationship between basal heart rate and IR injury. In addition, this study suggests that targeting Ca_v_1.3 channels could constitute a relevant target for reducing infarct size, since maximal heart rate dependent cardioprotective effect is already observed in *Ca_v_1.3^+/−^* mice.

## 1. Introduction

Cardiovascular diseases are the leading cause of morbidity and mortality worldwide in 2023. Among them, Acute Myocardial Infarction (AMI) is the number one killer and is responsible for a substantial fraction of fatalities of cardiovascular origin (1). Reperfusion is the only treatment available to reduce infarct size (2–4). There is an association between infarct size and the rate of mortality (5), making early reperfusion a critical therapeutic factor (“time is muscle”) (2). However, reperfusion therapy also induces detrimental effects referred to as lethal reperfusion injury (6–8). Reperfusion injury specifically induces cardiac cell death and significantly contributes to final infarct size as a second component of tissue damage besides ischemic damage (6, 7). To date, no pharmacologic or genetic therapy targeting ischemia-reperfusion (IR) injury and limiting the extent of the infarcted area is available (9). Clinical studies revealed that several factors, including aging and diabetes, are prone to change the integrity of survival pathways, impairing the effectiveness of recently proposed cardioprotective strategies (10, 11). Heart rate is another important determinant of cardiac pathology and mortality (2, 12, 13). Most ischemic episodes are triggered by an increase in heart rate that induces an imbalance between myocardial oxygen delivery and consumption (14). During cardiac ischemia, heart rate reduction prolongs the ventricular diastole augmenting the coronary perfusion, which improves oxygenation of the myocardium at rest and during exercise (15, 16). Development of new therapeutic strategies to reduce IR injury would constitute an important step towards reducing mortality and morbidity following AMI (17). In this context, appropriate heart rate reduction may limit the vulnerability of the myocardium during IR episodes (14).

Heart rate is generated in the sinoatrial node (SAN) by ion channels of the plasma membrane and ryanodine receptor-mediated intracellular Ca^2+^ release (18). Among ion channels, hyperpolarization-activated f-(HCN) channels underlying the “funny” current (*I_f_*) (19, 20), L-type Ca_v_1.3 (21) underlying L-type *I_Cav1.3_* Ca^2+^ current, are important determinants of heart rate (18, 22). Indeed, mice in which Ca_v_1.3 channels have been genetically ablated display slow SAN rhythm and ventricular bradycardia (21, 23). Besides ion channels potentiating SAN pacemaking, G-protein gated (GIRK4) K^+^ channels underlying cardiac acetylcholine-activated K^+^ current (*I_KACh_*) constitute an important mechanism to slow heart rate upon parasympathetic activity (24, 25). Mice lacking GIRK4 channels (*Girk4^−/−^*) display slight basal tachycardia and blunt cholinergic regulation of SAN pacemaking (25). The f-channel blocker ivabradine, which reduces heart rate was reported to decrease infarct size during an IR protocol through heart rate-dependent and independent mechanisms (26, 27). We hypothetized that, because of their implication in controlling heart automaticity, Ca_v_1.3 channels, beside f-channels, are potential pharmacologic targets for heart rate regulation during cardiac ischemia to limit reperfusion injury. We thus investigated the impact of heart rate modulation by inactivation of SAN Ca_v_1.3 or GIRK4 channels on IR injury in mice. Then, ivabradine was used as a heart rate reducing agent during IR injury and its effect compared to those mediated by genetic inactivation of Ca_v_1.3 or GIRK4.

## 2. Materials and methods

### 2.1. Animals

Wild-type (WT, Charles River laboratory) and transgenic mice harboring *Ca_v_1.3^−/−^*, *Ca_v_1.3^+/−^*, or *Girk4^−/−^* genotype on C57BL/6J genetic background were used in this study. Experiments were performed in accordance with the European Communities Council directive of November 1986 and conformed to the Guide for the Care and Use of Laboratory Animals” published by the US National Institutes of Health (NIH publication 8th Edition, 2011). All mice were maintained under environmentally controlled conditions (22 ± 2°C, 12 h light/12 h dark cycle) in the animal facility of the Institute. The surgical protocols were approved by the “Comité d’Ethique pour l’Expérimentation Animale Languedoc-Roussillon” (CEEA-LR) with the authorization number CE-LR-0814. For *ex vivo* experiments, the protocol “Study of physiological parameters and cardioprotection *ex vivo*” was approved by the SBEA (Structure Bien-être Animal) committee from the Institute according to the European directive 2010/63/EU and the French ministerial regulation of February 01, 2013. The “*n*” number of mice included in the study for each experiment is the “*n*” authorized by the ethics committee as being the minimal “*n*” number allowing a valid statistical analysis while respecting the 3R rule (Replacement, Reduction, Refinement) established in Europe.

### 2.2. *In vivo* surgical model of myocardial ischemia and reperfusion

Acute myocardial ischemia and reperfusion were induced in *Ca_v_1.3^−/−^*, *Ca_v_1.3^+/−^* and *Girk4^−/−^*, as well as in WT controls as previously described (28). Adult male mice (22–32 g) were anesthetized with an intramuscular injection of ketamine (25 mg/kg; Imalgène 500, Merial, France), xylazine (10 mg/kg; Rompum 2%, Bayer, France), and chlorpromazine (1.25 mg/kg; Largactil 5 mg/ml, Sanofi-Aventis, France). Mice were ventilated via tracheal intubation with a Harvard rodent respirator (tidal volume, 7.2 µl/g body mass; respiratory rate, 200 breaths per min). Body temperature was maintained between 36.8°C and 37°C via a thermoregulated surgical table connected to a rectal probe. One-lead surface electrocardiograms (ECG) were recorded by Ag/AgCl gel-coated electrodes (Unomedical) attached to the superior right and both inferior limbs of mice and connected to a standard one-lead ECG amplifier module (EMKA Technologies, Paris, France). The recordings were carried out from 10 min before ischemia (baseline) until the end of the experience. Mice were randomly enrolled in the control group [NaCl 0.9%, intraperitoneal (IP) injection] or ivabradine-injected group (6 mg/kg, IP, 30 min before ischemia). Then, after a second injection of ketamine (25 mg/kg) and xylazine (10 mg/kg), the chest was opened by a left lateral thoracotomy, and a reversible coronary artery snare occluder was placed around the left coronary artery. All animals underwent 40 min of ischemia followed by 60 min of reperfusion by loosening of the knot. At the beginning of reperfusion, a third administration of ketamine/xylazine was performed (same dose). At the end of reperfusion, the artery was reoccluded, phthalocyanine blue was injected into the left ventricular (LV) cavity and allowed to perfuse the non-ischemic portions of the myocardium, and the heart was removed for infarct size (IS) analysis.

### 2.3. *Ex vivo* model of regional ischemia-reperfusion

Adult male mice were anesthetized with an intraperitoneal dose of ketamine (100 mg/kg) and xylazine (10 mg/kg), followed by sodium pentobarbital (45 mg/kg; Ceva Santé Animale, France). An IP injection of heparin (7,500 IU/kg; Sanofi Aventis, France) was administered to avoid thrombus formation. A thoracotomy was performed and beating hearts were quickly removed and excised into ice-cold perfusion fluid. The aorta was cannulated under magnification and hearts were quickly mounted and perfused by the Langendorff method (29) on an isolated heart system apparatus (Isolated heart system, EMKA Technologies, France). The coronary circulation was perfused at 70 mmHg over a 1–4 ml/min flow range with modified Krebs-Henseleit buffer containing (mM): NaCl 116; KCl 5; MgSO_4_ 1.1; NaH_2_P0_4_ 0.35; NaHCO_3_ 27; CaCl_2_ 1.8 and glucose 10. The buffer was gassed with 95% O_2%_ and 5% CO_2_, giving a pH of 7.4 at 37°C. Before use, the buffer was initially passed through a 0.2 µm filter (Sarstedt, USA), and all perfusate delivered to hearts was filtered via an in-line 0.45 µm filter for removing microparticulates.

During the stabilization phase, a 7.0 prolene ligature (Johnson & Johnson Ethicon, Belgium) was placed around the left coronary artery 1–2 mm distal from where it emerges beneath the left atrium. The ends of the ligature were passed through an occluder tube, initially without tension. ECG was continuously recorded by Ag/AgCl electrodes placed on the right atrium and near the apex. Heart temperature was continuously assessed by a probe positioned on the base of the heart and recorded by using a TH-5 monitoring thermometer (Phymep, USA). Perfused hearts were then immersed in a water-jacketed bath and continuously maintained at 37°C. After a stabilization phase of 15 min (baseline), regional normothermic ischemia was then induced by tightening the suture with the occluder tube for 40 min. After ischemia, reperfusion was allowed by loosening the ligature and perfusing the heart for 60 min. For atrial pacing, a bipolar stimulating electrode was placed on the right atrium and pacing was started during the stabilization phase.

At the end of reperfusion, the artery was reoccluded, the hearts were removed from the perfusion system and phthalocyanine blue was injected through the aortic cannula to perfuse the non-ischemic portions of the myocardium, allowing the area at risk to be delineated.

### 2.4. Recording of coronary blood flow

During the whole *ex vivo* IR protocol, the coronary flow from the isolated heart system apparatus was continuously digitized at 0.1 Hz and recorded using an IOX data acquisition system (EMKA Technologies, France). Excel software (Microsoft, USA) was used to perform offline analysis of the data recorded. For each mouse, a mean value of maximal coronary flow (MaxF) was calculated every 10 min. Values during ischemia and reperfusion were normalized with respect to the pre-ischemia phase (baseline) and were expressed in percentages.

### 2.5. ECG recordings and analysis

For both *in vivo* and *ex vivo* models, signals were digitized continuously at 1 kHz and recorded using an IOX data acquisition system (EMKA Technologies, France). EcgAuto software (EMKA Technologies, France) was used to perform offline analysis of the data recorded. For each mouse the mean heart rate (HR) value during baseline, ischemia and reperfusion was calculated.

### 2.6. Infarct size assessment

Infarct sizes were measured using the technique developed by Fishbein et al. (30). Hearts from *in vivo* and *ex vivo* experiments were dissected and the LVs were sliced transversally into 1 mm-thick sections and incubated in a 1% (w/v) solution of 2.3.5-triphenyltetrazolium chloride (TTC; Sigma-Aldrich, St Louis, Mo) for 15 min at 37°C. After fixation in a 4% paraformaldehyde-phosphate-buffered solution, the slices were weighted, and each side was photographed with an Olympus camera. The ischemic risk area (unstained in blue) and the infarcted area (unstained by TTC) were outlined on each photograph and measured by planimetry using Image J (Scion corp., Frederick, MD). The area of each region was averaged from the photographs of each side for each slice, and multiplied by the weight of that tissue section. Infarct size was expressed as a percentage of the ischemic risk area (AR). Mice with an AR of less than 40 (in % of LV mass) and those greater than 60 were excluded from the study to ensure that infarct size values were compared in a pool of animals with the same infarct severity.

### 2.7. Statistical analysis

All values are expressed as mean ± SD. For comparisons between two groups, the Mann–Whitney non-parametric test was used. Multiple comparisons between groups were assessed by the Kruskal–Wallis nonparametric test followed by the Dunn's *post hoc* test or ANOVA (when the Normality Shapiro–Wilk test passed) or by the three-way ANOVA when considering three different variables (time, mutation and pacing). Values of *p* < 0.05 were accepted as statistically significant. The *p* values, indicated in the text, were noted in the figures as: ns for *p* > 0.05, * for *p* < 0.05, ** for *p* < 0.001, *** *p* < 0.01 and **** *p* < 0.0001. All data were analyzed with GraphPad Prism (GraphPad Software, San Diego, California, USA). A linear relationship between HR and IS was fitted by linear regression model using the least squares method (GraphPad Prism software) based on the minimization of the sum of squares of the vertical distances of the points from the line.

## 3. Results

### 3.1. Pharmacological inhibition of f-channels by ivabradine reduces infarct size upon IR condition *in vivo*

**Figure 1:**
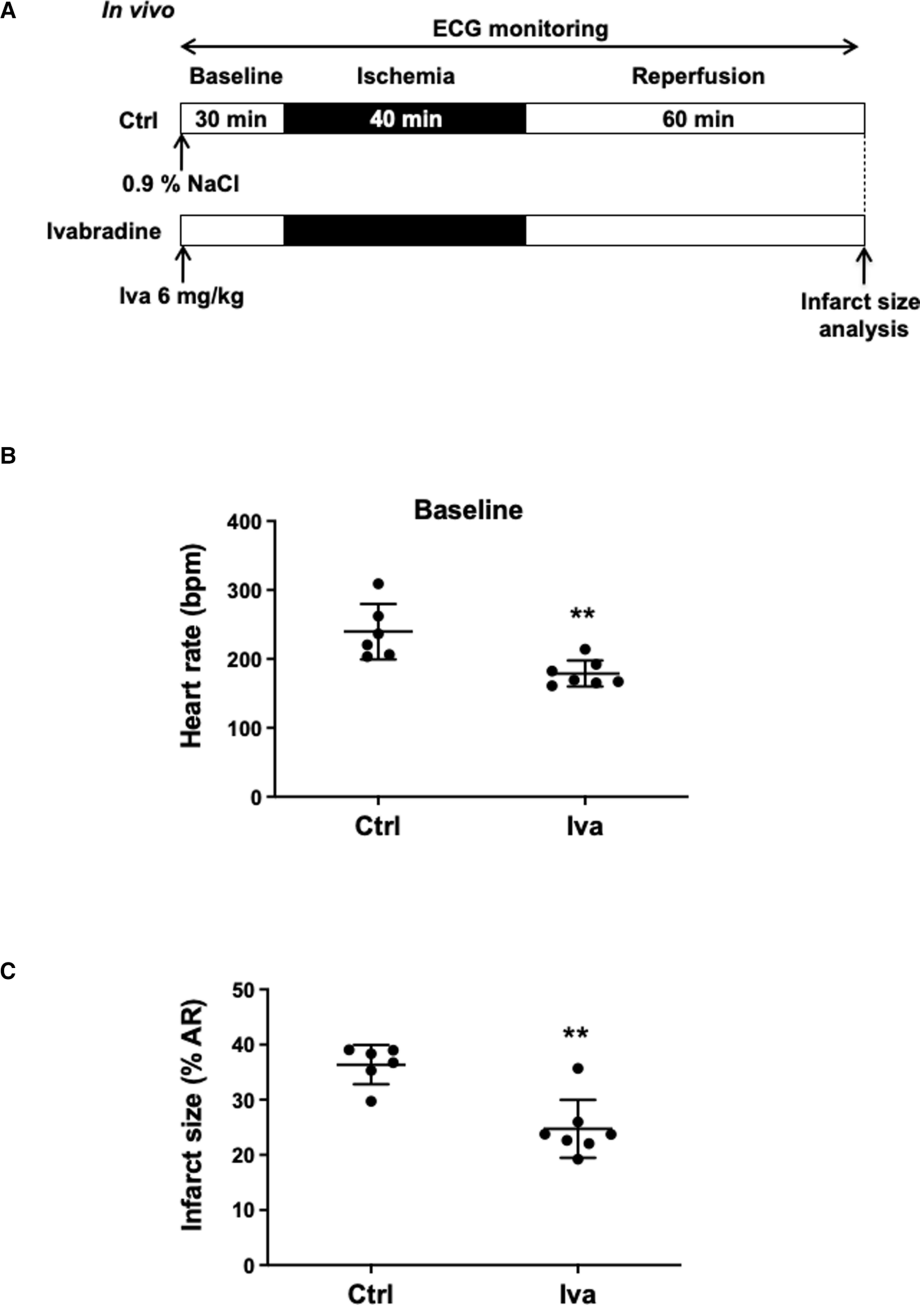
(**A**) *In vivo* experimental protocol applied to wild-type (WT) mice, consisting of 40 min of ischemia induced by ligation of the left coronary artery followed by 60 min of reperfusion. Mice were assigned to receive either 0.9% NaCl (Ctrl, *n* = 6) or Ivabradine (Iva, 6 mg/kg, *n* = 7), intraperitoneally 30 min before ischemia. ECG was recorded during the surgical protocol; (**B**) scatter dot plots and mean ± SD were plotted for baseline HR measured before ischemia in control mice (NaCl 0.9%) (*n* = 6) or Iva pre-treated mice (*n* = 7); (**C**) scatter dot plots and mean ± SD were plotted for infarct size (expressed as the percentage of the area at risk) and measured at the end of the surgical protocol in control (Ctrl, *n* = 6) and Iva pre-treated (Iva, *n* = 7) mice subjected to ischemia-reperfusion (IR) protocol; Statistical analysis was performed using the non-parametric Mann–Whitney test. Statistical significance is noted ** for *p* = 0.0047 for (**B,C**).

The hyperpolarization-activated current (*I_f_*) plays an important role in the generation of heart rate. Therefore, we first investigated the effects of the selective *I_f_* inhibitor ivabradine on IR injury (Figure 1A). Anesthetized WT mice were subjected to an *in vivo* surgery protocol. ECG was continuously recorded along the surgery protocol (Figure 1A; Supplementary Figure S1). In comparison with control WT mice (WT; 0.9% NaCl, IP, *n* = 6), ivabradine treated mice (WT Iva; 6 mg/kg; IP injection, 30 min before coronary ligation; *n* = 7) showed a significant decrease in both basal heart rate (179 ± 19 bpm vs. 240 ± 40 bpm, **p = 0.0047; 25%-reduction; Figure 1B) and infarct size (24.73% ± 5.25% vs. 36.37% ± 3.56%, **p = 0.0047; 32%-reduction; Figure 1C). The area at risk of ischemia (AR/LV mass) was similar among groups (*p*^ns ^= 0.9452; Supplementary Figures S2A).

### 3.2. Genetic ablation of L-type Ca_v_1.3 channels reduced infarct size upon IR *in vivo*

**Figure 2:**
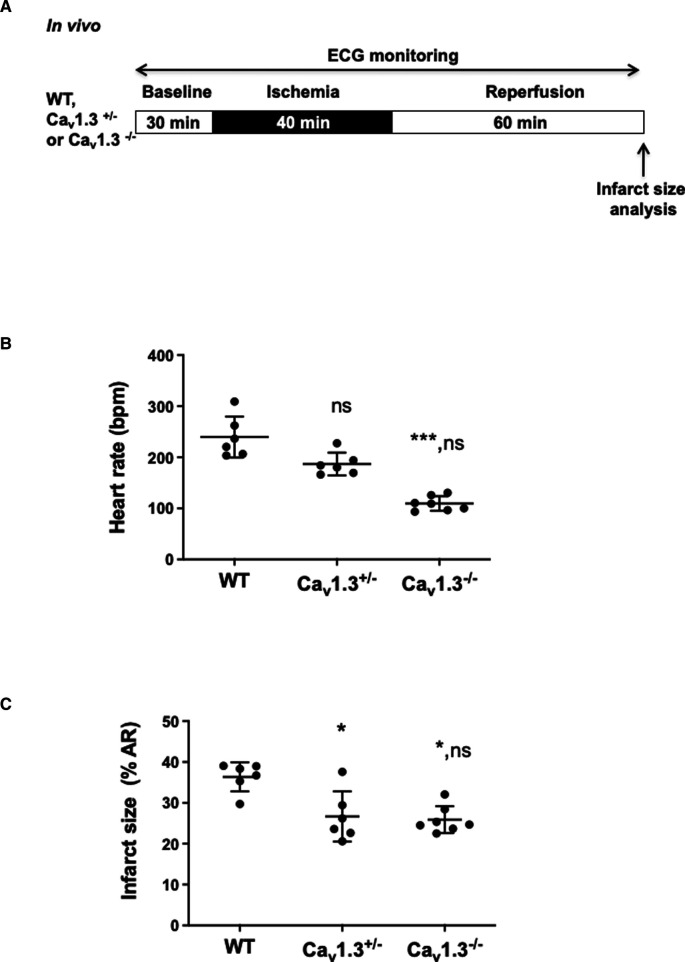
(**A**) *In vivo* experimental protocol applied to wild-type (WT), *Ca_v_1.3^+/−^* and Ca_v_*1.3^−/−^* mice, consisting in 40 min ischemia, induced by ligation of the left coronary artery, followed by 60 min of reperfusion. ECG was recorded during the surgical protocol; (**B**) scatter dot blots and mean ± SD were plotted for baseline HR measured before ischemia for WT mice (NaCl 0.9%) (*n* = 6), *Ca_v_1.3^+/−^* (*n* = 6) and *Ca_v_1.3^−/−^* (*n* = 7); (**C**) scatter dot plots of infarct size and means ± SD were plotted for all mice subjected to IR protocol. Non parametric Kruskal–Wallis test followed by Dunn's *post hoc* test was used to compare data among groups. ns is noted for *p* > 0.05, * for *p* < 0.05 and *** for *p* < 0.001 vs. control; ns in second place was noted for *p* > 0.05 vs. *Ca_v_1.3^+/−^*.

Because ivabradine was described to provide also heart rate-independent benefit in cardioprotection (31), we evaluated the cardioprotective effects of heart rate reduction obtained through genetic deletion of Ca_v_1.3 channels, a major contributor in the generation of diastolic depolarization. To this aim, anesthetized *Ca_v_1.3^+/−^* and *Ca_v_1.3^−/−^* mice were both subjected to the *in vivo* surgical protocol presented in Figure 2A with a continuous ECG recording along the protocol (Supplementary Figure S1). Figure 2B shows that heterozygous *Ca_v_1.3^+/−^* mice exhibit moderate heart rate reduction (22%) (187 ± 22 bpm, *n* = 6 for *Ca_v_1.3^+/^*^−^ vs. 240 ± 40 bpm, *n* = 6 for Ctrl; *p*^ns ^= 0.3714) that was associated with a 26.7%-decrease in infarct size (26.67% ± 6.16%, *n* = 6 for *Ca_v_1.3^+/−^* vs. 36.37% ± 3.56%, *n* = 6 for WT; p* = 0.0267). In homozygous *Ca_v_1.3^−/−^* mice (*n* = 7), characterized by a marked reduction (54%) of the basal heart rate in comparison to WT counterparts (*n* = 6) (109 ± 14 bpm, *n* = 7 for *Ca_v_1.3^−/−^* vs. 240 ± 40 bpm, *n* = 6 for Ctrl; p*** = 0.0004), a 28.8%-decrease in infarct size vs. WT was observed (25.90% ± 3.28%, *n* = 7 for *Ca_v_1.3^−/−^* vs. 36.37% ± 3.56%, *n* = 6 for WT; p* = 0.0190; Figure 2C). Area at risk of ischemia (AR/LV mass) was similar among groups (*p*^ns ^= 0.0511; Supplementary Figure S2B), indicating that all hearts were exposed *in vivo* to quantitatively similar ischemic challenge. These results suggest that moderate heart reduction induced by genetic ablation of one allele of Ca_v_1.3 channels exerted potent cardioprotective effect against IR injury.

### 3.3. Heart rate and infarct size *in vivo* displayed linear correlation

**Figure 3:**
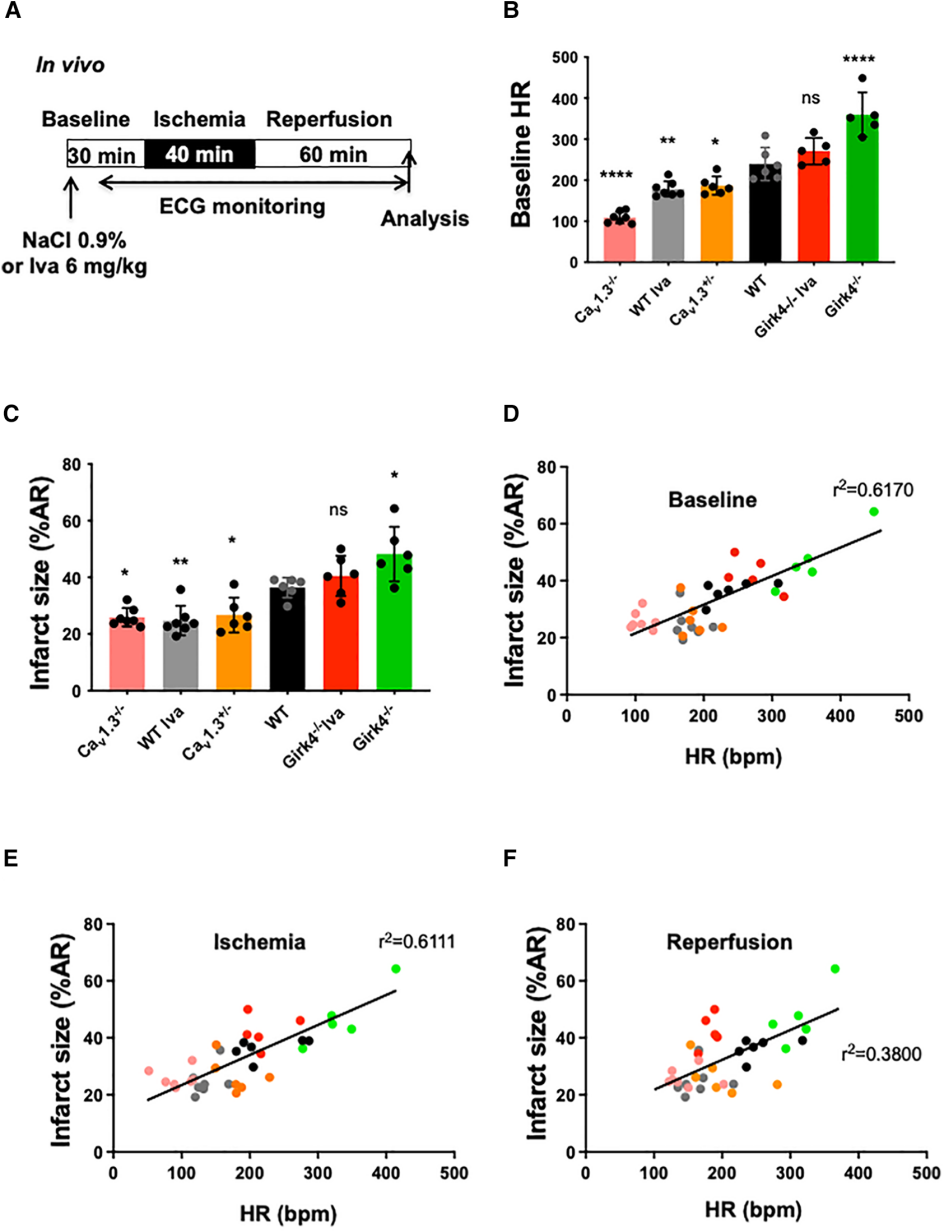
Correlation between infarct size and heart rate during ischemia-reperfusion in wild-type (WT) and mutant mice. (**A**) IR protocol was induced by reversible coronary ligation leading to 40 min ischemia followed by 60 min reperfusion. Ivabradine (6 mg/kg, IP route) was injected in WT (WT Iva, *n* = 7) and *Girk4^−/−^* (*Girk4^−/−^* Iva, *n* = 5) mice; (**B**) scatter dot plots and bars (mean ± SD) are presented for heart rates recorded at baseline in WT (*n* = 6) and in *Ca_v_1.3^+/−^* (*n* = 6), Ca_v_*1.3^−/−^* (*n* = 7) and *Girk4^−/−^* (*n* = 5) mutant mice, or WT Iva and *Girk4^−/−^* Iva. Colors represent particular mouse genotype or ivabradine treatment. Data were compared using ANOVA (normality Shapiro-Wilk test passed) and *p* values are given vs. WT: * is noted for *p* = 0.0282, ** for *p* = 0.0069, **** for *p* < 0.0001 and ns for *p* = 0.3557). (**C**) scatter dot plots and bars (mean ± SD) are presented for infarct size recorded at baseline in WT and mutant mice from different genotypes, or WT and *Girk4^−/−^* mice pre-treated with one bolus injection of ivabradine. Note that the lowest heart rate for *Ca_v_1.3^−/−^* hearts is associated with reduced infarct size, while moderate tachycardia in *Girk4^−/−^* mice is associated to increased infarct size compared to WT mice. Data were compared using ANOVA (normality Shapiro-Wilk test passed) and *p* values are given vs. WT; ** is noted for *p* = 0.0062, * for *p* = 0.0342 (*Ca_v_1.3^+/−^)*, *p* = 0.0151 (*Ca_v_1.3^−/−^)*, *p* = 0.0212 (*Girk4^−/−^) and* ns for *p* = 0.3355). A linear correlation between infarct size (in % of AR) and heart rate (bpm) during baseline (**D**) ischemia (**E**) and reperfusion (**F**) is shown. *R*^2^ values are noted on each graph.

In order to elucidate the correlation between heart rate and infarct size, we used genetically modified mice harboring different basal heart rates. We thus measured infarct size in *Ca_v_1.3^+/−^*, *Ca_v_1.3^−/−^* and *Girk4^−/−^* mice subjected to the same IR protocol (Figure 3A) in control conditions (WT) and after ivabradine (WT Iva; 6 mg/kg) treatment. Figure 3B shows variation in basal heart rate according to the different genotypes, with heart rate of *Ca_v_1.3^−/−^* being the lowest and that of *Girk4^−/−^* the highest among groups (Supplementary Figure S1). Mean heart rates of *Ca_v_1.3^−/−^*, *Ca_v_1.3^+/−^ and WT Iva* were lower than that of WT counterparts (109 ± 14 bpm, *n* = 6; p**** < 0.0001 for *Ca_v_1.3^−/−^*; 187 ± 22 bpm, *n* = 6; *p* = 0.0282 for *Ca_v_1.3^+/−^* and 179 ± 19 bpm, *n* = 7; p** = 0.0069 vs. 240 ± 40 bpm, *n* = 6, for WT). By contrast, mean heart rates from *Girk4^−/−^* and *Girk4^−/−^* Iva were higher compared to WT [360 ± 54 bpm, *n* = 5; p****^ ^< 0.0001 for *Girk4^−/−^* and 271 ± 32 bpm, *n* = 5; *p*^ns ^= 0.356 for *Girk4^−/−^* Iva vs. WT (240 ± 40 bpm, *n* = 6). Figure 3B]. Figure 3C shows infarct size analysis among the various genotypes and the clear correlation between infarct size and heart rate. *Girk4^−/−^* mutant mice exhibit the largest (47.26% ± 4.67%, *n* = 5) and *Ca_v_1.3^−/−^* the lowest (25.9% ± 3.28%, *n* = 7) infarct sizes. A moderate heart rate reduction in *Girk4*^−/−^ mice induced by a bolus injection of ivabradine (40.5% ± 7.09%, *n* = 7) yielded infarct sizes that were in between those recorded in WT and *Girk4 ^−/−^* mice.

Of note, higher *r*^2^ values of the linear regression were obtained for heart rate values recorded at baseline and during ischemia (*r*^2 ^= 0.6170 and *r*^2 ^= 0.6111, respectively; Figures 3D,E) than those recorded during reperfusion (*r*^2 ^= 0.3800; Figure 3F). Irrespective of heart rates recorded at baseline and the genotype of mice tested, the heart rate was significantly decreased during ischemia, while increased during reperfusion (Figure 3C; Supplementary Figure S1). The relationship between heart rate recorded at baseline and infarct size was preserved for heart rates recorded during ischemia (Figures 3D,E) and, to a lesser extent, during reperfusion (Figure 3F). AR/LV mass measured for each heart was similar among groups (*p*^ns ^= 0.0506) (Supplementary Figure S3).

### 3.4. Atrial pacing *ex vivo* negated the cardioprotective effect of heart rate modulation

**Figure 4:**
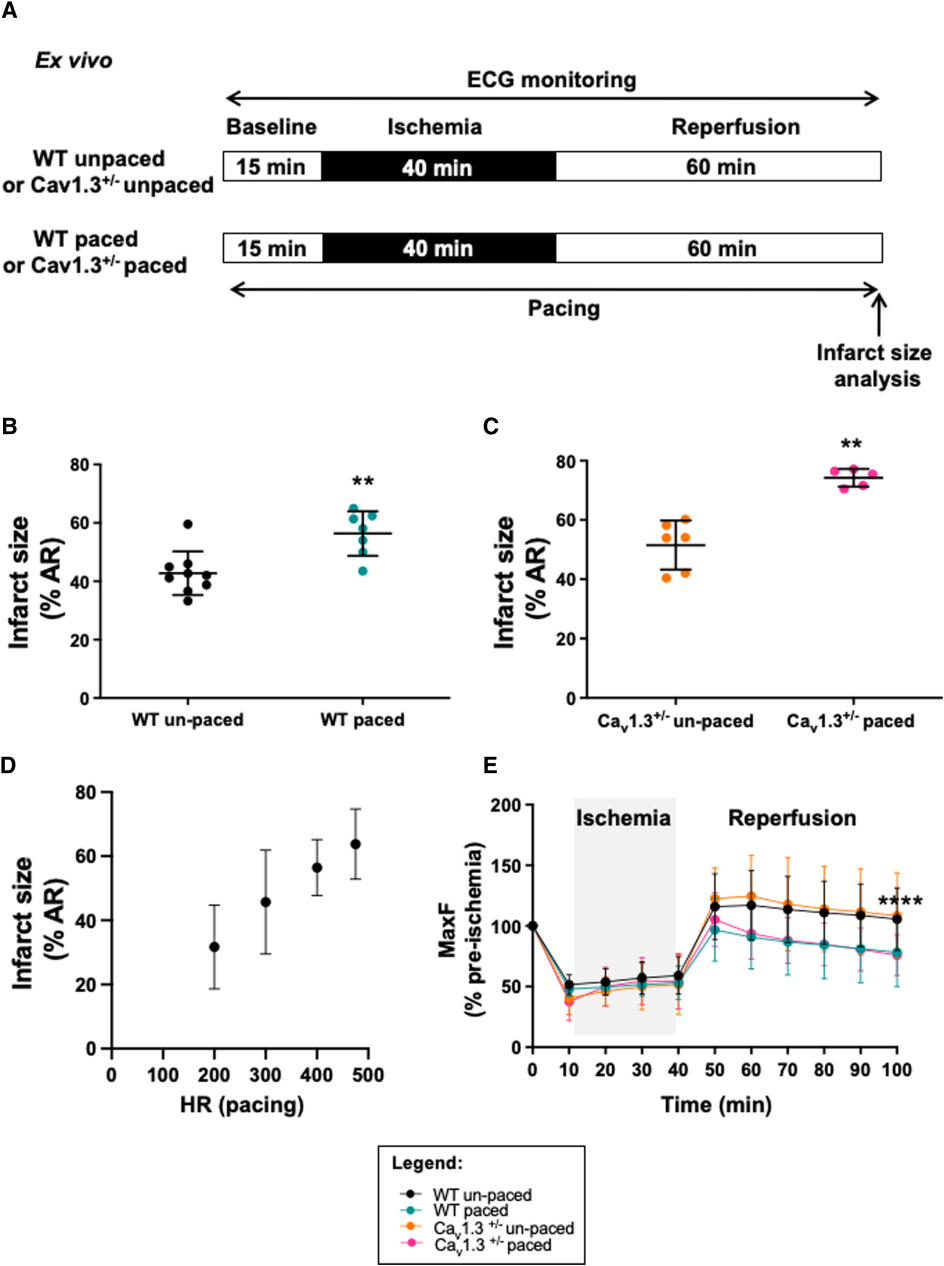
Infarct size and heart rate during e*x vivo* ischemia-reperfusion of langendorff perfused WT and *Ca_v_1.3^+/−^* mouse hearts with or without arial pacing. Protocol of induction of ischemia-reperfusion injury and pacing is shown in (**A**). Scatter dot plot and mean ± SD are presented for infarct size values (expressed as the percentage of area at risk) for paced un-paced (*n* = 6) and paced (*n* = 5) Cav1.3^-/-^ hearts (**B**) as well as for paced (*n* = 7) and un-paced (*n* = 9) WT hearts (**C**). (**D**) relationship between infarct size and pacing frequency in WT hearts; (**E**) maximum coronary flow (MaxF) was expressed as a percentage of pre-ischemia value during e*x vivo* ischemia-reperfusion in un-paced WT (black symbol and line) and *Ca_v_1.3^+/−^* (orange color), paced WT (blue) and paced *Ca_v_1.3^+/−^* (pink) hearts. The grey area indicates the ischemic period. Statistical analysis was performed using the three-way ANOVA, considering the time, the pacing and the *Ca_v_1.3^−/+^* mutation as variables. Results show that variability was due to the time factor (p**** < 0.0001) and the pacing (p**** < 0.0001) with a significant interaction (p** = 0.0075). However, the effect of the mutation of Ca_v_1.3 was not significant (*p*^ns ^= 0.9841). If we consider only the period of reperfusion, only the pacing impacts the results (p**** < 0.0001).

Since the infarct size correlated with heart rate irrespective of the genotype *in vivo*, we investigated whether increase in heart rate prevented cardioprotection. To this aim, atrial pacing *ex vivo* was performed to increase heart rate in the absence of the vagus nerve influence (Figure 4). Hearts underwent the same protocol as *in vivo* through the implementation of a reversible ligation on the left coronary during 40 min ischemia followed by 60 min reperfusion (Figure 4A). In the atrial-paced group, hearts were placed at 475 bpm. Since *Ca_v_1.3^−/−^* hearts *ex vivo* exhibit complex atrioventricular conduction block (32), which could interfere in experiments of atrial pacing, we used hearts from *Ca_v_1.3^+/−^* associated with strong cardioprotection. *Ca_v_1.3^+/−^* hearts with a mean heart rate of 341 ± 20 bpm (*n* = 6) were paced at 475 bpm (*n* = 5), representing a 39%-increase. Infarct size in paced hearts was 44%-increased (74.22% ± 2.99%, *n* = 5 in paced vs. 51.52% ± 8.32%, *n* = 6 in un-paced Ca_v_1.3^+/−^; p** = 0.0043; Figure 4B). Same experiments were performed in C57Bl6 WT mice in order to overcome the influence genetic modifications. Heart rate at baseline was 398 ± 50 bpm in the WT group (*n* = 9) and upon atrial pacing (20% increment in heart rate), infarct size was increased by 32% in those hearts (56.33% ± 7.45%, *n* = 7 vs. 42.78% ± 7.45%, *n* = 9; p** = 0.0052; Figure 4C). Consistently, the relationship between infarct size and heart rate was maintained *ex vivo* by pacing WT hearts to lower heart rate values including 200, 300 and 400 bpm. Figure 4D shows a linear correlation established between infarct size and heart rate in the range of 200–475 bpm (Supplementary Figure S5).

Since heart rate reduction prolongs the ventricular diastole allowing to augment the coronary perfusion, we recorded the coronary flow during *ex vivo* experiments (Figure 4E; Table 1). Our results show that coronary flow dropped during the ischemic phase (≈50%) in WT and *Ca_v_1.3^+/−^* paced or un-paced hearts. However, during reperfusion, the coronary flow of un-paced WT and *Ca_v_1.3^+/−^* hearts recovered and stabilized above 100% of the control baseline level, whereas the flows in paced hearts of both genotypes did not fully recover after stabilization and was reduced by 25% compared to the un-paced condition (p**** < 0.0001).

**Table 1:** Mean, SD and *n* values are presented for the maximal coronary flow (maxF), calculated every 10 min and expressed as a percentage of the data recorded at baseline (stabilization phase). Data were obtained during *ex vivo* experiments in WT and *Ca_v_1.3^+/−^* hearts, with or without pacing (475 bpm).

		WT			WT pacing			Ca_v_1.3^+/−^			Ca_v_1.3^+/−^ pacing		
Phase	Time (min)	Mean	SD	n	Mean	SD	n	Mean	SD	n	Mean	SD	n
Stabilization	0	100	0	9	100	0	7	100	0	5	100	0	7
Ischemia	10	52	8	9	48	6	7	40	14	5	48	6	7
	20	54	11	9	50	6	7	46	12	5	50	6	7
	30	57	13	9	52	10	7	50	19	5	52	10	7
	40	59	15	9	53	14	7	52	24	5	53	14	7
Reperfusion	50	116	27	9	97	26	7	123	25	5	97	26	7
	60	117	28	9	91	26	7	125	34	5	91	26	7
	70	114	27	9	87	27	7	118	39	5	87	27	7
	80	111	26	9	84	28	7	114	35	5	84	28	7
	90	109	26	9	81	28	7	112	36	5	81	28	7
	100	106	26	9	78	28	7	108	35	5	78	28	7

Taken together, these results demonstrate positive correlation between heart rate and infarct size *ex vivo* and suggest that the cardioprotective effect associated to a decreased heart rate might be related to a better coronary circulation during reperfusion.

## 4. Discussion

In this study we have investigated the relationship between heart rate and infarct size using a protocol of IR injury *in vivo*. We found that infarct size is closely correlated with heart rate recorded during the pre-ischemic and ischemic phases in WT and mutant mice tested. Genetic ablation of one allele of L-type Ca_v_1.3 channels led to moderate heart rate reduction and a reduced infarct size *in vivo* when compared to WT counterparts. Increased heart rate via atrial pacing *ex vivo* abolished both the cardioprotective effect of heart rate reduction induced by genetic ablation of Ca_v_1.3 and improvement of the post-ischemic coronary flow observed at a lower heart rate. Taken together, our data show, for the first time, that heart rate *per se* is a major predictor of IR injury: the lower is heart rate, the greater being cardioprotection. In conclusion, our results indicate positive correlation between heart rate and infarct size both *in vivo* and *ex vivo* in the absence of vagal stimulation. Furthermore, our data suggest that targeting of Ca_v_1.3 channels can constitute a new potential therapeutic strategy to reduce IR injury and infarct size.

Heart rate is a vital parameter assessed at admission in all patients with acute myocardial infarction. Several prognostic studies have shown that resting heart rate is predictive of poor outcome in patients and heart rate at discharge is predictive of long-term outcome in patients with coronary artery disease (33). In this context, β-blockers have shown beneficial effects in terms of reduction of mortality after AMI (34, 35). They are widely prescribed for cardiovascular disease because their heart rate reduction effect. These drugs are effective for restoration of contractility in left ventricular dysfunction (36). However, despite these beneficial effects, they can provide intolerance and exercise limitation in patients because of their negative inotropic effect. We have shown in a previous study that β-blocker treatment abolishes in human cells the high frequency-induced upregulation of human cardiac Ca_v_1.2-driven calcium currents, a physiological mechanism involved in the Bowditch “staircase” allowing an adaptation to exercise and stress upon β-adrenergic regulation (37). In addition, β-blockers also exert α-adrenergic vasoconstriction, responsible for deleterious effects on myocardial coronary blood flow and contractile function (38). These findings highlight the importance of selective heart rate reduction through modulation of SAN automaticity in the absence of concomitant negative inotropism (14, 16). Pharmacological targeting of ion channels involved in pacemaker activity thus constitutes a promising avenue for protecting the myocardium from ischemic injury. In this respect, clinical trials have shown the beneficial effects of selective heart rate reduction by ivabradine, in the context of coronary artery disease and heart failure (39, 40). As suggested recently by Baka et al. cardioprotection by ivabradine could be beneficial in the management of COVID-19-related cardiovascular complications including myocarditis, acute coronary syndrome, cardiogenic shock or cardiac dysautonomia (41). However, clinical studies have shown that treatment with high doses of ivabradine involves an increased risk of cardiovascular mortality, infarction and atrial fibrillation (42, 43). These results underscore the need to investigate new potential ion channels as targets to develop new therapies aimed to selectively control heart rate without adverse effects on the myocardium and prognosis of patients.

Ca_v_1.3 channels underlying *I_Cav1.3_* current constitute with *I_f_* the predominant mechanisms in the generation of diastolic depolarization (21, 32, 44), while GIRK4 channels underlying *I_KACh_* constitute both tonic and phasic brakes of diastolic depolarization and heart rate (25, 45). In addition, our previous study about heart rate determination by Cav1.3 and f-channels indicates that these channels possibly act independently in pacemaker activity because Ca_v_1.3 knockout and ivabradine reduced heart rate by similar percentages in freely-moving mice (39). This is in line with our results in the present study. Indeed, f-channel blockade and Ca_v_1.3 knockout lead to comparable infarct sizes, supporting our hypothesis that cardioprotection mediated by Ca_v_1.3 targeting is primarily due to heart rate reduction, at least under our experimental conditions. Furthermore, Ca_v_1.3 channels are not expressed in the adult ventricle (21) and are not involved in excitation-to-contraction coupling in the adult heart (46). Ca_v_1.3 channels thus constitute an important candidate, in addition to *I_f_*, to obtain slowing of heart rate without concomitant negative inotropism. Consequently, we have compared cardioprotection conferred by pharmacological inhibition of *I_f_* to that obtained upon genetic ablation of Ca_v_1.3 and GIRK4 channels.

Pharmacological heart rate reduction by ivabradine effectively reduced the heart rate and the infarct size in control WT mice (Figure 1), thus validating our *in vivo* mouse model of IR for studying the effects of genetic ablation of ion channels involved in pacemaking. Our results show that genetic ablation of Ca_v_1.3, or GIRK4 channels regulates the infarct size in close correlation with heart rate recorded in three phases considered: baseline, ischemia and reperfusion. Importantly, genetic targeting of Ca_v_1.3 channels and pharmacologic inhibition of *I_f_* reduced infarct size, similarly, suggesting comparable efficacy in controlling IR injury (Figure 3). Consequently, when the correlation between heart rate and infarct size is considered in the three phases, genetic ablation of Ca_v_1.3 compares to ivabradine, suggesting that selective pharmacological targeting of Ca_v_1.3 could induce at least as much cardioprotection as ivabradine. Consistently with heart rate being an important factor in determining cardioprotection against IR injury, similar heart rates obtained by ivabradine infusion, or genetic ablation of Ca_v_1.3 channels yielded comparable infarct sizes. We noticed that despite *Ca_v_1.3^−/−^* mice presented with lower heart rates than *Ca_v_1.3^+/−^* mice, deletion of the two Ca_v_1.3 alleles did not significantly reduce the infarct size in comparison to heterozygous *Ca_v_1.3^+/−^* mice (Figure 2), possibly as a result of a maximal saturating protective effect of such mechanism in our model. Indeed, given the results obtained with ivabradine, we cannot exclude that infarct size value of 25% of the area at risk constitutes the lowermost limit in size that can be obtained by genetic or pharmacological targeting of ion channels involved in the generation of SAN diastolic depolarization. However, other hypotheses can be put forward to explain the equivalent infarct sizes between *Ca_v_1.3^+/−^* heterozygous and *Cav1.3^−/−^* homozygous hearts have increased degree of remodeling secondary to sporadic atrioventricular block found in this genotype (32, 45). Such a remodeling may limit indirectly cardioprotection in relation to absolute heart rate; A second, non-exclusive, reason could reside in an intrinsic physiological limit of cardioprotection—around 29% in our model—that can be obtained by heart rate reduction during the ischemic phase. The additional effect of ivabradine on reducing infarct size (reaching 32%-reduction) could be explained by previously reported rate-independent cardioprotection effect (26, 27). In this regard, it is worth to note that the 50% reduction in heart rate observed in anesthetized *Ca_v_1.3^−/−^* mice exceeds bradycardia observed *in vivo* in freely moving *Cav1.3^−/−^* mice. This could be related to the cardiodepressant effect of xylazine-ketamine-based anesthesia, which would tend to exacerbate the effects on heart rate of total genetic ablation of Ca_v_1.3 in cardiac pacemaker activity, an effect that would not translate into overt cardioprotection compared to *Ca_v_1.3^+/−^* hearts. Bradycardia during the acute phase of myocardial infarction is correlated to early cardiovascular mortality. In their study, Salwa et al. demonstrated a J-curve for the mortality as a function of heart rates suggesting that bradycardia was associated to worsen injury within the myocardial tissue (47). Future study using selective pharmacological Ca_v_1.3 blockade may help explain these discrepancies.

The cardioprotective effect observed in *Ca_v_1.3^+/−^* hearts was abolished when atrial pacing was applied to compensate the lowering effect of Ca_v_1.3 inactivation expressed in the pacemaker of *Ca_v_1.3^+/−^* mice, clearly showing that cardioprotection was related to heart rate reduction, since Ca_v_1.3 channels are not expressed in the adult ventricle (Figure 4). These observations reinforce our conclusion that heart rate is a major determinant of cardioprotection conferred by genetic ablation of Ca_v_1.3 channels. Our data indicate that, similar to that reported for ivabradine (14, 26), improvement of coronary flow at the reperfusion phase underlies rate-dependent cardioprotection (Figure 4F). In line with this hypothesis, rapid pacing abolished the improvement of coronary perfusion in both WT and *Ca_v_1.3^+/−^* hearts. In this context, previous work showed that part of the cardioprotective effect of ivabradine is not dependent on its heart rate reduction effect (26, 27). For Ca_v_1.3 mutant hearts, atrial pacing *ex vivo* negated the cardioprotective effect of the genetic ablation of one allele of the Ca_v_1.3 gene. These results obtained in isolated perfused hearts suggest that cardioprotection observed in *Ca_v_1.3^+/−^* was dependent on its heart rate reduction effect.

## Data Availability

The original contributions presented in the study are included in the article/**Supplementary Material**, further inquiries can be directed to the corresponding author.

## References

[1] O'Gara PT Kushner FG Ascheim DD Casey DE Jr Chung MK de Lemos JA 2013 ACCF/AHA guideline for the management of ST-elevation myocardial infarction: a report of the American college of cardiology foundation/American heart association task force on practice guidelines. J Am Coll Cardiol. (2013) 61(4):e78–140. 10.1016/j.jacc.2012.11.019 23256914

[2] Maroko PR Kjekshus JK Sobel BE Watanabe T Covell JW Ross J Jr Factors influencing infarct size following experimental coronary artery occlusions. Circulation. (1971) 43(1):67–82. 10.1161/01.CIR.43.1.67 5540853

[3] Ginks WR Sybers HD Maroko PR Covell JW Sobel BE Ross J Jr. Coronary artery reperfusion. II. Reduction of myocardial infarct size at 1 week after the coronary occlusion. J Clin Invest. (1972) 51(10):2717–23. 10.1172/JCI107091 5056664 PMC332972

[4] Maroko PR Libby P Ginks WR Bloor CM Shell WE Sobel BE Coronary artery reperfusion. I. Early effects on local myocardial function and the extent of myocardial necrosis. J Clin Invest. (1972) 51(10):2710–6. 10.1172/JCI107090 5056663 PMC332971

[5] Gibbons RJ Valeti US Araoz PA Jaffe AS. The quantification of infarct size. J Am Coll Cardiol. (2004) 44(8):1533–42. 10.1016/j.jacc.2004.06.071 15489082

[6] Jennings RB Sommers HM Smyth GA Flack HA Linn H. Myocardial necrosis induced by temporary occlusion of a coronary artery in the dog. Arch Pathol. (1960) 70:68–78. PMID: 14407094

[7] Garcia-Dorado D Piper HM. Postconditioning: reperfusion of “reperfusion injury” after hibernation. Cardiovasc Res. (2006) 69(1):1–3. 10.1016/j.cardiores.2005.11.011 16337162

[8] Yellon DM Hausenloy DJ. Myocardial reperfusion injury. N Engl J Med. (2007) 357(11):1121–35. 10.1056/NEJMra071667 17855673

[9] Ibanez B Heusch G Ovize M Van de Werf F. Evolving therapies for myocardial ischemia/reperfusion injury. J Am Coll Cardiol. (2015) 65(14):1454–71. 10.1016/j.jacc.2015.02.032 25857912

[10] Ferdinandy P Schulz R Baxter GF. Interaction of cardiovascular risk factors with myocardial ischemia/reperfusion injury, preconditioning, and postconditioning. Pharmacol Rev. (2007) 59(4):418–58. 10.1124/pr.107.06002 18048761

[11] Ferdinandy P Hausenloy DJ Heusch G Baxter GF Schulz R. Interaction of risk factors, comorbidities, and comedications with ischemia/reperfusion injury and cardioprotection by preconditioning, postconditioning, and remote conditioning. Pharmacol Rev. (2014) 66(4):1142–74. 10.1124/pr.113.008300 25261534

[12] Jouven X Empana JP Schwartz PJ Desnos M Courbon D Ducimetiere P. Heart-rate profile during exercise as a predictor of sudden death. N Engl J Med. (2005) 352(19):1951–8. 10.1056/NEJMoa043012 15888695

[13] Hjalmarson A Gilpin EA Kjekshus J Schieman G Nicod P Henning H Influence of heart rate on mortality after acute myocardial infarction. Am J Cardiol. (1990) 65(9):547–53. 10.1016/0002-9149(90)91029-6 1968702

[14] Heusch G. Heart rate in the pathophysiology of coronary blood flow and myocardial ischaemia: benefit from selective bradycardic agents. Br J Pharmacol. (2008) 153(8):1589–601. 10.1038/sj.bjp.0707673 18223669 PMC2438254

[15] Indolfi C Ross J Jr. The role of heart rate in myocardial ischemia and infarction: implications of myocardial perfusion-contraction matching. Prog Cardiovasc Dis. (1993) 36(1):61–74. 10.1016/0033-0620(93)90022-6 8100637

[16] DiFrancesco D Camm JA. Heart rate lowering by specific and selective *I*(*f*) current inhibition with ivabradine: a new therapeutic perspective in cardiovascular disease. Drugs. (2004) 64(16):1757–65. 10.2165/00003495-200464160-00003 15301560

[17] Hausenloy DJ Erik Botker H Condorelli G Ferdinandy P Garcia-Dorado D Heusch G Translating cardioprotection for patient benefit: position paper from the working group of cellular biology of the heart of the European society of cardiology. Cardiovasc Res. (2013) 98(1):7–27. 10.1093/cvr/cvt004 23334258

[18] Mangoni ME Nargeot J. Genesis and regulation of the heart automaticity. Physiol Rev. (2008) 88(3):919–82. 10.1152/physrev.00018.2007 18626064

[19] DiFrancesco D. The role of the funny current in pacemaker activity. Circ Res. (2010) 106(3):434–46. 10.1161/CIRCRESAHA.109.208041 20167941

[20] Mesirca P Alig J Torrente AG Muller JC Marger L Rollin A Cardiac arrhythmia induced by genetic silencing of ‘funny’ (f) channels is rescued by GIRK4 inactivation. Nat Commun. (2014) 5:4664. 10.1038/ncomms5664 25144323 PMC4207211

[21] Mangoni ME Couette B Bourinet E Platzer J Reimer D Striessnig J Functional role of L-type Cav1.3 Ca2+ channels in cardiac pacemaker activity. Proc Natl Acad Sci U S A. (2003) 100(9):5543–8. 10.1073/pnas.0935295100 12700358 PMC154381

[22] Mesirca P Torrente AG Mangoni ME. Functional role of voltage gated Ca(2+) channels in heart automaticity. Front Physiol. (2015) 6:19. 10.3389/fphys.2015.00019 25698974 PMC4313592

[23] Platzer J Engel J Schrott-Fischer A Stephan K Bova S Chen H Congenital deafness and sinoatrial node dysfunction in mice lacking class D L-type Ca^2+^ channels. Cell. (2000) 102(1):89–97. 10.1016/S0092-8674(00)00013-1 10929716

[24] Wickman K Nemec J Gendler SJ Clapham DE. Abnormal heart rate regulation in GIRK4 knockout mice. Neuron. (1998) 20(1):103–14. 10.1016/S0896-6273(00)80438-9 9459446

[25] Mesirca P Marger L Toyoda F Rizzetto R Audoubert M Dubel S The G-protein-gated K+ channel, IKACh, is required for regulation of pacemaker activity and recovery of resting heart rate after sympathetic stimulation. J Gen Physiol. (2013) 142(2):113–26. 10.1085/jgp.201310996 23858001 PMC3727310

[26] Heusch G Skyschally A Gres P van Caster P Schilawa D Schulz R. Improvement of regional myocardial blood flow and function and reduction of infarct size with ivabradine: protection beyond heart rate reduction. Eur Heart J. (2008) 29(18):2265–75. 10.1093/eurheartj/ehn337 18621770

[27] Kleinbongard P Gedik N Witting P Freedman B Klocker N Heusch G. Pleiotropic, heart rate-independent cardioprotection by ivabradine. Br J Pharmacol. (2015) 172(17):4380–90. 10.1111/bph.13220 26076181 PMC4556475

[28] Roubille F Combes S Leal-Sanchez J Barrere C Cransac F Sportouch-Dukhan C Myocardial expression of a dominant-negative form of daxx decreases infarct size and attenuates apoptosis in an in vivo mouse model of ischemia/reperfusion injury. Circulation. (2007) 116(23):2709–17. 10.1161/CIRCULATIONAHA.107.694844 18025529

[29] Langendorff O. Untersuchungen am überlebenden säugethiererhenzen. Archiv für die gesammte phisiologie des menschen under thiere. Bonn. (1895) 61:2911–332. 10.1007/BF01662056

[30] Fishbein MC Meerbaum S Rit J Lando U Kanmatsuse K Mercier JC Early phase acute myocardial infarct size quantification: validation of the triphenyl tetrazolium chloride tissue enzyme staining technique. Am Heart J. (1981) 101(5):593–600. 10.1016/0002-8703(81)90226-X 6164281

[31] Heusch G Kleinbongard P. Ivabradine: cardioprotection by and beyond heart rate reduction. Drugs. (2016) 76(7):733–40. 10.1007/s40265-016-0567-2 27041289

[32] Baudot M Torre E Bidaud I Louradour J Torrente AG Fossier L Concomitant genetic ablation of L-type Cav1.3 (α1D) and T-type Cav3.1 (α1G) Ca^2+^ channels disrupts heart automaticity. Sci Rep. (2020) 10(1):18906. 10.1038/s41598-020-76049-7 33144668 PMC7642305

[33] Jensen MT Pereira M Araujo C Malmivaara A Ferrieres J Degano IR Heart rate at admission is a predictor of in-hospital mortality in patients with acute coronary syndromes: results from 58 European hospitals: the European hospital benchmarking by outcomes in acute coronary syndrome processes study. Eur Heart J Acute Cardiovasc Care. (2018) 7(2):149–57. 10.1177/2048872616672077 27694532

[34] Hjalmarson A Elmfeldt D Herlitz J Holmberg S Malek I Nyberg G Effect on mortality of metoprolol in acute myocardial infarction. A double-blind randomised trial. Lancet. (1981) 2(8251):823–7. 10.1016/S0140-6736(81)91101-6 6116950

[35] Gottlieb SS McCarter RJ Vogel RA. Effect of beta-blockade on mortality among high-risk and low-risk patients after myocardial infarction. N Engl J Med. (1998) 339(8):489–97. 10.1056/NEJM199808203390801 9709041

[36] Nagatsu M Spinale FG Koide M Tagawa H DeFreitas G Gt C Bradycardia and the role of beta-blockade in the amelioration of left ventricular dysfunction. Circulation. (2000) 101(6):653–9. 10.1161/01.CIR.101.6.653 10673258

[37] Piot C Lemaire S Albat B Seguin J Nargeot J Richard S. High frequency-induced upregulation of human cardiac calcium currents. Circulation. (1996) 93(1):120–8. 10.1161/01.CIR.93.1.120 8616918

[38] Heusch G Baumgart D Camici P Chilian W Gregorini L Hess O alpha-adrenergic coronary vasoconstriction and myocardial ischemia in humans. Circulation. (2000) 101(6):689–94. 10.1161/01.CIR.101.6.689 10673263

[39] Fox K Ford I Steg PG Tendera M Robertson M Ferrari R. Heart rate as a prognostic risk factor in patients with coronary artery disease and left-ventricular systolic dysfunction (BEAUTIFUL): a subgroup analysis of a randomised controlled trial. Lancet. (2008) 372(9641):817–21. 10.1016/S0140-6736(08)61171-X 18757091

[40] Swedberg K Komajda M Bohm M Borer JS Ford I Dubost-Brama A Ivabradine and outcomes in chronic heart failure (SHIFT): a randomised placebo-controlled study. Lancet. (2010) 376(9744):875–85. 10.1016/S0140-6736(10)61198-1 20801500

[41] Baka T Repova K Luptak I Simko F. Ivabradine in the management of COVID-19-related cardiovascular complications: a perspective. Curr Pharm Des. (2022) 28(19):1581–8. 10.2174/1381612828666220328114236 35345992

[42] Fox K Ford I Steg PG Tardif JC Tendera M Ferrari R. Ivabradine in stable coronary artery disease without clinical heart failure. N Engl J Med. (2014) 371(12):1091–9. 10.1056/NEJMoa1406430 25176136

[43] Martin RI Pogoryelova O Koref MS Bourke JP Teare MD Keavney BD. Atrial fibrillation associated with ivabradine treatment: meta-analysis of randomised controlled trials. Heart. (2014) 100(19):1506–10. 10.1136/heartjnl-2014-305482 24951486 PMC4174120

[44] Torrente AG Mesirca P Neco P Rizzetto R Dubel S Barrere C L-type Cav1.3 channels regulate ryanodine receptor-dependent Ca^2+^ release during sino-atrial node pacemaker activity. Cardiovasc Res. (2016) 109(3):451–61. 10.1093/cvr/cvw006 26786159

[45] Mesirca P Bidaud I Briec F Evain S Torrente AG Le Quang K G protein-gated IKACh channels as therapeutic targets for treatment of sick sinus syndrome and heart block. Proc Natl Acad Sci U S A. (2016) 113(7):E932–41. 10.1073/pnas.1517181113 26831068 PMC4763776

[46] Matthes J Yildirim L Wietzorrek G Reimer D Striessnig J Herzig S. Disturbed atrio-ventricular conduction and normal contractile function in isolated hearts from Cav1.3-knockout mice. Naunyn Schmiedebergs Arch Pharmacol. (2004) 369(6):554–62. 10.1007/s00210-004-0940-7 15146309

[47] Salwa P Gorczyca-Michta I Wożakowska-Kapłon B. The relationship between admission heart rate and early prognosis in patients with ST-elevation myocardial infarction. Kardiol Pol. (2015) 73(3):177–82. 10.5603/KP.a2014.0171 25179485

