# Supplementary material for: Heart rate reduction after genetic ablation of L-type Cav1.3 channels induces cardioprotection against ischemia-reperfusion injury
Source: Front Cardiovasc Med. 2023 Aug 1;10:1134503. doi: 10.3389/fcvm.2023.1134503 (PMC10429177; doi:10.3389/fcvm.2023.1134503)
Supplement: Supplementary file 1 [file Datasheet1.docx]

**Heart rate reduction after genetic ablation of L-type Ca_v_1.3 channels induces cardioprotection against ischemia-reperfusion injury**

Delgado-Betancourt V. et al,

**Supplemental figures**

**
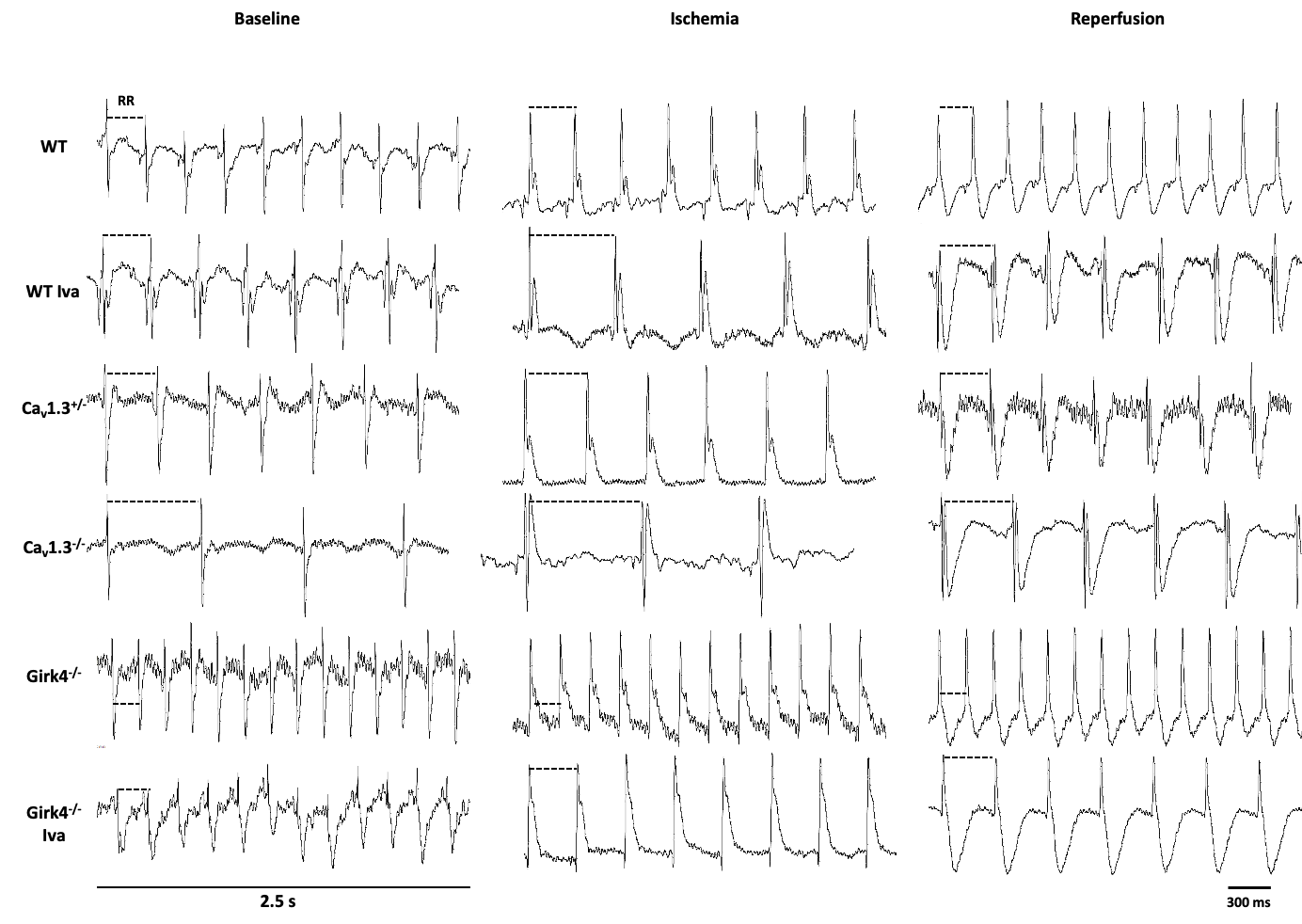
**

**Supplemental Figure 1:** Representative ECG recordings of anesthetized mice from different genotypes undergoing the protocol of ischemia-reperfusion *in vivo*. For each heart, the tracings recorded during baseline, ischemia and reperfusion periods are shown. The dotted lines represent the RR interval that was considered for heart rate measurement.

**
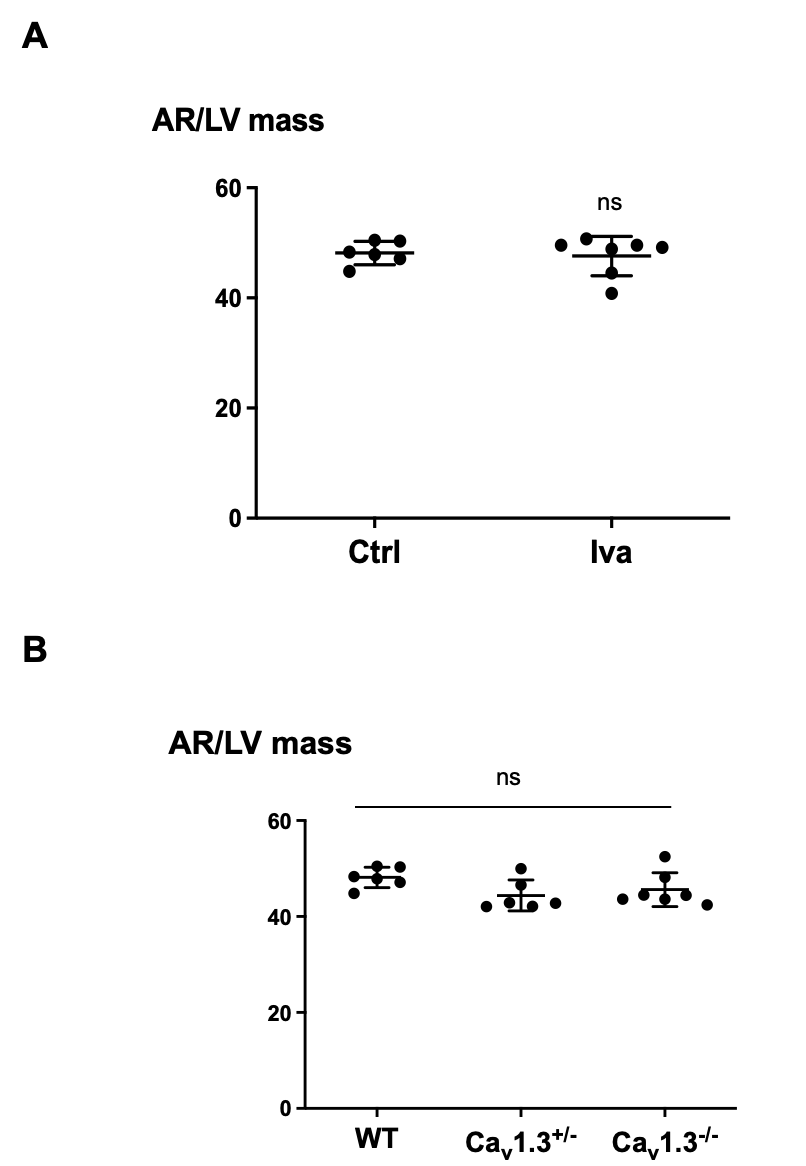
**

**Supplemental Figure 2: Area at risk measured in the study groups of mice subjected to IR *in vivo.***

**A:** Scatter dot blots and mean ± SD shows the AR values (expressed in % of the LV mass) obtained for each heart subjected *in vivo* to IR injury in control condition (Ctrl, n=6) or after Ivabradine (Iva, 6 mg/kg; n=7) treatment. Data were compared using the non-parametric Mann-Whitney test. ns was noted for p=0.9452; **B:** Scatter dot blots and means ± SD were plotted for AR/LV mass obtained for each heart subjected *in vivo* to IR injury in wild-type (WT, n=6), *Ca_v_1.3^+/-^* (n=6) and *Ca_v_1.3^-/-^* (n=7). Data were compared using the non-parametric Kruskal-Wallis test followed by the Dunn’s post-test. ns was noted for p= 0.0511.

**
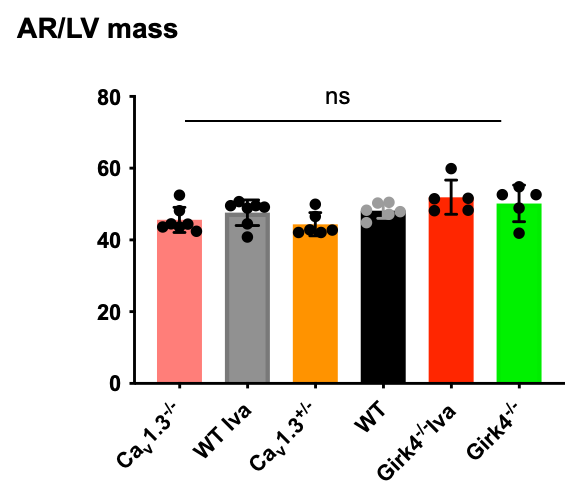
**

**Supplemental Figure 3: Area at risk measured in all the study groups of mice subjected to IR *in vivo.***

**A:** Scatter dot blots and bars represent the values and mean ± SD obtained for the AR (expressed in % of the LV mass) measured for each heart group of hearts after the *in vivo* experiments. There was no statistical difference among group ,showing that all mice were subjected to similar injury (ns is noted for p=0.0506).

**
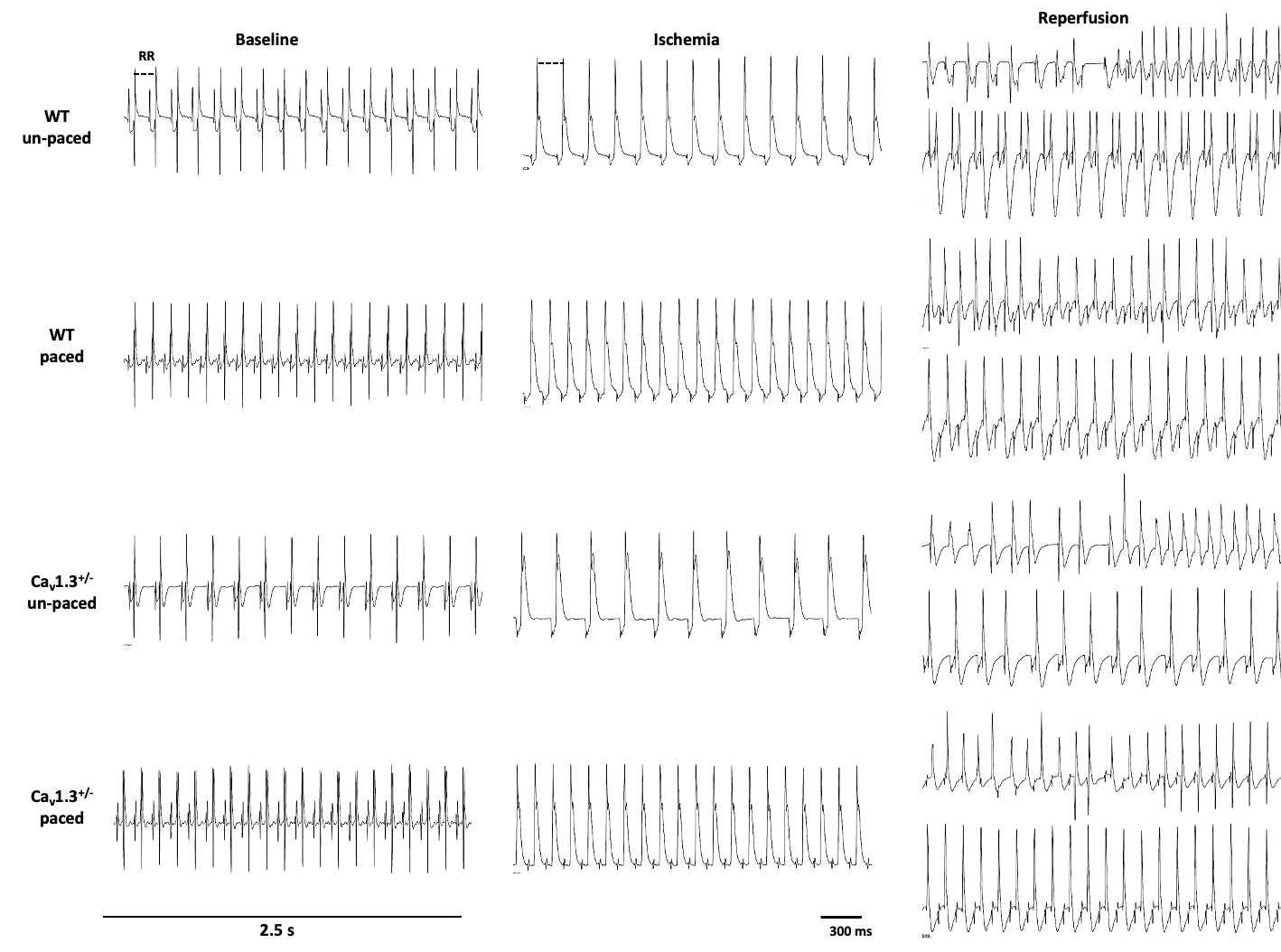
**

**Supplemental Figure 4:** Representative ECG recordings of anesthetized mice from WT and *Ca_v_1.3^+/-^* genotypes undergoing the protocol of ischemia-reperfusion *in vivo* with pacing at 475 bpm (paced) or not (un-paced). For each heart, the tracings recorded during baseline, ischemia and reperfusion periods are shown. The dotted lines represent the RR interval that was considered for heart rate measurement.


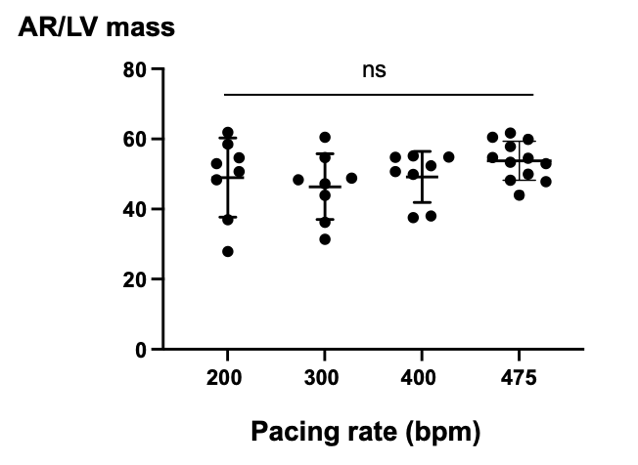


**Supplemental Figure 5: Area at risk measured in the study groups of mice subjected to IR *ex vivo* with and without pacing.**

**A:** Scatter dot blots and mean ± SD shows the AR values (expressed in % of the LV mass) obtained for each heart subjected *ex vivo* to IR injury in control condition under pacing at various rates: 200 (n=8), 300 (n=8), 400 (n=8) et 475 bpm (n=12) Data were compared using the non-parametric Kruskal-Wallis test followed by the Dunn’s post-test. ns was noted for p= 0.3405.
